# The Interplay of Infidelity, Sexuality, and Religiosity in the Discourse of Mixed-Orientation Marriages: A Discursive Psychological Analysis

**DOI:** 10.3389/fpsyg.2022.784675

**Published:** 2022-04-08

**Authors:** Mohd Asyraf Zulkffli, Radzuwan Ab. Rashid, Mohammad Affiq Kamarul Azlan, Hanita Hanim Ismail

**Affiliations:** ^1^Faculty of Language Studies and Human Development, Universiti Malaysia Kelantan, Bachok, Malaysia; ^2^Faculty of Languages and Communication, Universiti Sultan Zainal Abidin, Kuala Terengganu, Malaysia; ^3^Faculty of Education, National University of Malaysia, Bangi, Malaysia

**Keywords:** gay, Muslims, marriage, Islam, infidelity, Malaysia, discursive psychology

## Abstract

This research examines the complex interplay of religiosity, sexuality, and infidelity. We adopted a case study approach in this research, and discourse was made central to the analysis. There were two participants; both identified as homosexuals. One participant, Fahrin, is married while the other, Muzz, is divorced at the time of the interview. The participants were subjected to an in-depth, semi-structured interview to gauge their experiences, perceptions, opinions, beliefs, and thoughts on their sexuality, Islamic faith as well as relationship with their spouses. The data were then transcribed and analyzed using the Discursive Action Model (DAM) and Discursive Psychology (DP) frameworks. From the analysis, two overarching themes were identified. They are (1) the allocation of blame and accountability and (2) the participants’ attachment to their Islamic identity. The discussion then revolves around the societal role that pressures gay men to marry women and contextualizes the position of Islam on Homosexuality.

## Introduction

The establishment of Lesbian, Gay, Bisexual, Transgender (LGBT)’s rights as part of human rights has seen unprecedented success over the year, i.e., the number of countries legalizing same-sex marriage has grown to over 30 countries (Masci et al., 2019), and acceptance of LGBT rights globally is also at an all-time high (Poushter and Kent, 2020). Even so, rejection of LGBT remains strong in several communities all over the world. This rejection is mainly rooted in religious affiliation, especially among the Muslims (Habib, 2010), Jews (Irshai, 2018), as well as Christians. In the Unites States, African Americans (Sherkat et al., 2010) and Latinos (Ellison et al., 2011) are prominently more reluctant to affirm LGBT ideals due to their affiliation to their Christian belief. The influence a religion could have on an individual’s life could not be understated. This could not be more evident than when looking at research that addresses the issue of LGBT people of faiths where oftentimes they would choose to forgo their same-sex attraction in order to keep their religiosity intact (Jerome, 2013; Shah, 2018; Zulkffli and Rashid, 2019; Avishai, 2020). For Muslims, due to the entrenched heteronormativity and heterosexism of the society (Sarac, 2014; Zulkffli et al., 2021) they reside in, a number of these individuals would choose to marry women to fit in Boellstorff (2005), and this is the aspect that will be the focus of this study.

The phenomenon of Gay Men in Straight Marriages (GMiSM) in the context of this research setting, Malaysia, could perhaps be summed up through a vignette reported as a cover story in one of the country’s main national papers. The story, aptly titled “*Dilema Suamiku Gay*” (Malay for The Dilemma of Having a Gay Husband), chronicled the story of a woman who found out that the man she had been married to for 3 years was attracted to the same sex. The story detailed how even though the husband seemed like a good Muslim, the husband never had sex with Sarah, and it was implied that the husband was promiscuous with other men. The article ended with commentary from a religious figure who emphasized the *haram* (forbidden) status of homosexuality and how glorifying it would bring upon divine punishment from Allah to the society (Utusan Malaysia, 2019).

This vignette encapsulates the all-too-often scenario about the phenomenon of GMiSM in the context of Malay-Muslim Society. From the vignette, we can see how the three aspects—religiosity, sexuality, and infidelity feature prominently even in such a short story. For a newspaper, article to run a front-page story on an issue that is considered taboo in a conservative society like Malaysia where sodomy and “perverted” sexual acts are still punishable with up to 20 years of imprisonment and whipping (see Malaysian Penal Code 1998, Section 377A and 377B); where efforts to promote LGBT rights are actively thwarted by the authority (Devaraj, 2011; Ellis-Petersen, 2018; Su-Lyn, 2018), indicates that the gravity of this phenomenon has reached a critical point. Over the years, this feature story is not the only instance where this taboo issue leaks into the public spheres; other articles of similar veins have also appeared in popular media, e.g., mStar (2018) reported on how wives of gay husbands often come to know about their husbands’ infidelity with same-sex partners through conversations in their phones while myMetro (2016) reported on “sexual problems” such as homosexuality and transgenderism as being one of the factors for divorce among Malaysian couples from the year 2012 to 2014.

The case of GMiSM poses serious consequences, especially toward the wives. Extensive reports have been found on physical and psychological trauma (Hays and Samuels, 1989; Smith and Allred, 1990; Cheng, 2016) as well as health risks related to sexually transmitted disease (Kanter et al., 2011; Klaar, 2012) faced by wives of GMiSM. For the gay men, on the other hand, face difficulty in reconciling their sexuality even after marriage and are likely to cheat on their wives with other men (Kissil and Itzhaky, 2014; Zack and Ben-Ari, 2018). The risk would be further compounded due to the reclusive nature of LGBT in the Malaysian context, and thus, it is absolutely imperative that this phenomenon be explored further to provide a nuanced understanding of this issue in light of the local context. This is where this research comes into play. We adopt a case study approach with Discursive Psychology (DP) as an analytical lens in elucidating the cases presented in this paper. The focus of this research is on how the GMiSM participants construct their discourse vis-à-vis the notion of blame and accountability, as well as look into how their identity as Muslims GMiSM is constructed and negotiated. The notion of accountability in this research will look into how the subjects address their infidelity as well as the dissonance between their Islamic faith and their sexuality.

## Literature Review

The subjects of homosexual and Bisexual men who enter heterosexual marriages and their homosexual-heterosexual relationships have been extensively studied since the late 20th century when the field of sexuality was receiving more attention, and the LGBT movement was starting to gain momentum. Though instances of gay men marrying women are mostly reclusive, its prevalence is significant (Ben-Ari and Adler, 2010). Buxton (2001), through her work as a researcher in the field as well as director of Straight Spouse Network, a support group for women who are or were married to gay or bisexual men, gave a conservative estimate of two million men and women who are or were married to the opposite gender. The issue is mainly studied in the context of clinical psychology, where implications of the studies would be helpful in couple therapy as well as therapy for Gay and Bisexual Husbands and their wives.

Reflecting the typical hetero-centric hegemony of the time, research from the Western world often cited societal and religious pressures as reasons for gay men to marry women (Coleman, 1982; Ortiz and Scott, 1994). Abrahamic faiths (Judaism, Christianity, and Islam) hold staunch opposition to homosexuality and thus, for many gay men of these faiths, marriage is seen as a “cure” to their homosexuality (Coleman, 1982; Kissil and Itzhaky, 2014; Zulkffli and Rashid, 2016). Gay men also have a strong desire to build a family so that they can conform to their society’s heteronormative norms. This is especially the case if they belong to a conservative society regardless of their religion. This phenomenon is also especially pronounced in China (Cheng, 2016). Chinese society is often viewed as “hypermasculine”; sons are greatly valued because they would carry the family name. Hence, sons are greatly pressured to marry so they can fulfil their filial duty. China’s one-child policy also means that the pressure is even more intensified, as a family would only have that one child to continue their family legacy. As a result, more gay men enter straight marriages just to conform to societal pressure. So widespread is the phenomenon that there is even a specific term to describe wives of gay men in Chinese, which is “Tongqi.” Unlike their Western counterparts, the Chinese Tongqi often face verbal, mental, and physical abuse. Tang et al. (2019) reported on how the patriarchal Chinese society enables such abuses. The husbands will often threaten to take custody of the children if the wives dare ask for a divorce. Society in general also does not take kindly if the wives air the issue about their husbands. Instead of sympathy and support, these women were often blamed for their husbands’ homosexuality. In research of Boellstorff (2005) of gay men in Indonesia, one gay man who was married to a woman refused to divorce his wife after his wife found out about his boyfriend. He also weaponized his Islamic faith by citing that only men have the right to initiate divorce in Islam. This, however, is only a half-truth. In Islam, women do have the right to initiate divorce, but the process is far more complicated for women than it is for men. Women have to engage with the Islamic courts, producing proofs of their husbands’ wrongdoings (a process which could take years), while for men, they could just end their marriage without due reasons.

Gay Men in Straight Marriages also significantly affect the spouses. Wives of GMiSM in study of Buxton (2006) study faced significant trauma when their husbands’ sexuality and adulterous behavior were made known to them. As their husbands delve deeper into their adulterous homosexual relations, the emotional and sexual connections with the husbands start to fade away. The continued rejection deprived them of intimacy and emotional fulfillment. This “starvation” even drives some of them to become promiscuous themselves. They also experienced an acute sense of isolation as the reclusive nature of their situation meant that not many people could relate and sympathize with their predicament. The shame of being perceived as inadequate and the reason for their husbands’ behavior also keep them from seeking support. Similar consequences are also reported by Klaar (2012). In addition to isolation, wives of gay husbands’ participants of Klaar (2012) also had their sense-of-self shattered as they blamed themselves for the unraveling of their relationships. They also reflected on how they were aware of their husbands’ sexuality but refused to acknowledge it. The denial is a way for them to cope as they struggle to keep the façade of their marriage. Considering the profound stake the phenomenon could cause, it is imperative that more research is done to better understand the experience of GMiSM and what compels them to engage in infidelity.

Upon disclosure of the husbands’ sexuality, the couples’ marriage would usually end in its dissolution. However, there are instances where gay and bisexual husbands and their straight wives choose to work on their relationship and keep their marriage. Bisexual participants in research of Edser and Shea (2002) cope with the problem by practicing effective and constant communication so that the wives are fulfilled emotionally and sexually. The husbands’ constant reassurance that they would remain faithful to their wives also helped keep their marriage intact. In research of Kissil and Itzhaky (2014) of GMiSM in an orthodox Jewish community, couples treat their relationships which are devoid of intimacy and romantic relationship, by treating their familial relationship as part of the larger societal institution. Family units were expected to contribute to the community by taking part in religious institutions as well as raising well-educated children.

Conversely, couples in research of Adler and Ben-Ari (2016) went to the extreme end by keeping their relationship open. The gay husbands would keep having sexual encounters with men, and their wives were fine with it. One husband would even relay to his wife about those encounters. The wife then would be turned on by his stories and that had the effect of spicing up their sex life. In turn, Benack and Swan (2016) leveled their criticisms toward the research community for their failure to acknowledge the unconventional and evolving conception homosexual-heterosexual couples have on their marriage and romantic relationship. They also posited that the traditional conception of marriage, where marriage is prescribed as a monogamist, and with someone one has sexual attraction to, is not the only one that exists. Some gay men willingly enter straight marriage because they love the women they are marrying, and such marriage is equally valid. A similar conception is also found among wives of gay men in study of Adler and Ben-Ari (2017). The women were willing to reconstruct the perception of their marital relationship so that the heteronormative, monogamous ideal was done away with to make way for marital relationships that were more open.

## Methodology

This research involved two participants who identify as gay, Muslim and is or had married a woman. They were subjected to an in-depth, semi-structured interview focusing on their experiences revolving around their identity as gay Muslims, their relationship with their wives, as well as their homosexual behavior before and during their marriages. Both participants were in their 30 s. They are given pseudonyms, Fahrin and Muzz. Their profiles are as follows:

### Fahrin

Fahrin has been married for 5 years, with two children. He lives separately with his wife since she works in a different city. He was sexually active with men before he married his wife and had a relationship with a man for 7 years before his marriage. After his marriage, he still has sexual relations with men “once every 2, 3 months.” He acknowledges that homosexual relationship is a sin and practice the five daily prayers and fasting diligently.

### Muzz

Muzz is a divorcee with a child. His marriage lasted for 5 years. He has been sexually active since he was 10 years old, and before his marriage, he had a relationship of 4 years with a man. Like Fahrin, he also had a long-distance relationship with his wife. During his marriage, he also had sexual relations with other men “once every 3, 4 months.” The marriage ended after he revealed his sexual behavior to his wife during a fight. His wife was willing to go on with the marriage provided that he promised to stop having sex with other men. He could not, so they got a divorce. He was not a practicing Muslim but decided to repent and practice Islam seriously after he got infected with HIV. He got infected after his divorce.

Case study approach is employed in this study as it enables an in-depth investigation of individuals, thus allowing researchers to gain valuable insights into the lives of the subjects being studied. Case study is also a robust framework for the topic of this study as it centers the analysis around the perspectives of the subjects being directly in the experience themselves. A descriptive case study typically utilizes one or two instances of an event to show what a situation is like, and it serves primarily to make the unfamiliar familiar for the audience of the topic of discussion (Colorado State University, 2020). Undoubtedly, this is the sole intention of this study, which is to comprehend the notion of being a homosexual in a straight marriage.

To capture the intricacies of the two cases of GMiSM above, we employed an in-depth, semi-structured interview as recommended by Marrelli (2007). Interview promotes participation as participants are directly involved in providing information, having the liberty in giving their interpretation of events, and having the advantage of being the center of attention. We also adopted “memoing” in data collection, as suggested by Naghmeh et al. (2015), to help clarify, arrange, and develop ideas throughout the study. We also carefully followed the rigorous steps prior, during, and after the interview, such as ensuring that the recording equipment worked well, interviewing at a comfortable location, and systematically recording our data. These steps are important in ensuring that our data are accurately interpreted according to the context of the participants and the situations.

Discursive Action Model (DAM; Edwards and Potter, 1992) was adapted as the theoretical lens guiding the analysis of this research. Discursive Psychology (DP) and DAM are potent in exploring themes related to sensitive issues. DAM’s emphasis on Accountability, Discourse as Actions, and Management of Stake and Interest in talks is effective in providing nuanced insights to this otherwise elusive subject. DP and DAM have been proven useful in the exploration of various themes such as teachers’ engagement in social media (Rashid, 2017); discourse between family members and therapists (Patrika and Tseliou, 2016); and youth epistemic rights in slide decks by LGBTQ+ youth groups (Uttamchandani and Lester, 2020). The concern may arise where proponents of DP suggest that this model be used to study conversations in naturally occurring setting only and not for interview data (Edwards, 2005). To address this concern, in line with the guideline on how to conduct interview research (Potter and Hepburn, 2005), we provide as much context as possible in our analysis; as well as treat the interviewer’s lines of questioning as part of our interview data so that their talks are scrutinized just as critically as the interviewers’ responses. The interview was conducted in the Malay language. Hence, the verbatim transcripts were translated by a professional service with the explicit instruction that the translation is done so that not only the meaning is translated as closely as possible to its source language but also ensure that the stylistics and intricacies of the source language are captured as well. In this sense, the employment of DP and DAM as tools to analyze the data in this qualitative research is not only apt, but they are also potent in capturing the nuanced and contextualized elements in our subjects’ discourse and thus, allow us to unearth illuminating insight regarding the phenomenon.

Professing their homosexual identity amidst the country’s homophobic law and social structure could bring about serious legal and social implications to the participants. Hence, we actively took steps to ensure the privacy and safety of our participants are protected. For instance, the audios of the interview recording were kept in a password-protected external hard drive during the process of transcription and were promptly deleted once the transcriptions were made as recommended by Groenewald (2004). The information which could potentially expose the participants’ identities within the transcriptions was also erased. Paper of Speer and Potter (2000) which addresses the issue of heterosexist bias in discourse, was also referred to as we extrapolate our data. This is crucial in ensuring that our research does not contribute to prejudiced talk or homophobic treatment toward the LGBT community.

## Findings

In this section, we present our extrapolation of data in two overarching themes. The discourse between the interviewer and the two participants would be made central to our analysis. Furthermore, in line with the case study and DP frameworks, the analysis is heavily contextualized to capture the essence of talks as accurately as possible.

### The Allocation of Blame and Accountability

Both participants explicitly admitted that they cheated on their wives. Hence, for this section, we will look at how these men present their discourses in this light and relate their discourse to DAM’s notion of accountability (Edwards and Potter, 1992). In the interview, both Fahrin and Muzz were asked about their sexual relations with other men. In excerpt 1, Fahrin responded to the interviewer’s question about whether he had sexual relations prior to as well as during his marriage.

#### Excerpt 1

**Table 1:** 

1.	Interviewer:	So, before marriage, have you had sexual relationship with men?
2.	Fahrin:	I would be lying if I said no, yes, I had.
3.	Interviewer:	Even after marriage, do you still do that?
4.	Fahrin:	When we were first married, I could hold myself from meeting men.
5.		That is for like six months or so. Maybe it’s the wife factor as well,
6.		because it’s like she does not really pay me any attention and
7.		does not understand me. So I start back my old behavior.
8.	Interviewer:	What do you mean by she does not really pay attention to you?
9.	Fahrin:	Means, like if I am back from work. Come back at 9 already, want
10.		to eat, she is already asleep. So I want to talk and everything, not
11.		that I expect much attention but at least.. it means if it is like that,
12.		might as well I do not marry. I feel like that when she acts like that, starts to feel
13.		lonely again so I start my old behavior again. It is
14.		just me. I cannot speak for my friends because I do not know.

In Excerpt 1, we can see how Fahrin blamed his wife for his infidelity. That much is obvious. What is even more striking however, is the manner in which he did so “Maybe it’s the wife factor as well” [line 5]. In DAM, talk is considered as an action. Thus, such the assertion regarding the wife’s culpability has the effect of justifying his action of cheating on his wife. The rest of his talk in Excerpt 1 also works toward that. He employed List (Edwards and Potter, 1992) by listing what his wife did not do to please him, “she does not really pay me any attention and does not understand me” [lines 6 and 7]. In Contrast (Edwards and Potter, 1992) to his wife’s negligence of him, he otherwise framed his expectations as reasonable “not that I expect much attention” [lines 11 and 12]. Furthermore, he also asserted that he held himself from having sex with men “for six months or so” [line 5] as a way to show that he had done his part in keeping himself from betraying his marriage.

This notion that it is his wife who ought to bear the culpability for his cheating behavior is further reiterated in a later part of the interview.

#### Excerpt 2

**Table 2:** 

1.	Interviewer:	How do you feel towards your wife?
2.	Fahrin:	Do I love my wife? Well, when it comes to feeling *mmm* yea I care
3.		for her but it’s not to the extent that I love her. Because sometimes
4.		relationship if it is real, it’s about loving each other, right. But
5.		perhaps I- have not loved her to that extent. I merely care for her.
6.		Just merely-how shall I say this *ek*? Maybe if- if I were with a man,
7.		maybe I will have that feeling of care and love, right. Because
8.		maybe she does not show- aaa… how shall I say this- aaa… efforts
9.		to make me love her. To love and care for her, she does not do
10.		things to make me love her.
11.	Interviewer:	So, she does not show her emotion is it?
12.	Fahrin:	Maybe she does not follow- for me she does not- how shall I say
13.		this *ek*- she does not follow what I want. I’m a bit particular because
14.		sometimes I want this and that, I want this thing to be done like this,
15.		so there are a lot of things she cannot follow so this makes it difficult.
16.	Interviewer:	What are the examples of things you want?
17.	Fahrin:	For example, *ala* just simple things like what- like doing the laundry
18.		it should be done like this. Or like folding the clothes, it should be
19.		done like that, these are the examples. Because she- my wife- she
20.		is more- like what *ek*- she’s not really good in doing chores. Doing
21.		household chores I mean. Usually for people like me, she should
22.		please through things like these. When she can do those things, I would be like oooh- I’d love her. When I need to teach her those
23.		things, it would be a bit difficult.

In this excerpt, the talk revolves around Fahrin’s feeling toward his wife. It is apparent that he feels like it is his wife’s fault that he does not love her. Here, Fahrin employs Systematic Vagueness (Edwards and Potter, 1992) when he lists all the things his wife does that cause him not to love her. The vague reporting of what his wife does, i.e., she does not put in “efforts to make me love her” [lines 8 and 9], and she fails to do “things to make me love her” [line 10] has the effect of making his discourse not susceptible to be refuted. Only after being asked by the interviewer to explicitly give examples [line 16] of what he means did he cites a reason for not loving his wives, i.e., he is not happy with the way his wife does “laundry” [line 17] and “folding the clothes” [line 18]. It also ought to be noted that these reasons are rather trivial when contrasted with the gravity of him not loving his lawfully wedded wife. Besides, it is also telling how Fahrin centers his entire talk around his needs “sometimes I want this and that, I want this thing to be done like this” [line 14]; and “for people like me, she should please through things like these” [lines 21 and 22].

Another notable aspect of Fahrin’s discourse is his insistence that it is his wife that is to blame for his infidelity rather than his sexuality. In Excerpt 1, we can see how he puts forward such a notion, and it is further reinstated in Excerpt 2 of his wife’s inadequacy to please him. He only alludes about his sexuality only once “Maybe if- if I were, with a man, I will have that feeling of love and care right” [lines 6 and 7] and even so, such notion is not elaborated further by Fahrin instead he again quickly shifts the focus of his talk toward his wife. Thus, by not putting forward the notion that his sexuality may be the reason for his infidelity, Fahrin again manages to deflect accountability of his action.

Next, we will look at how Muzz talks about the nature of his relationship with his then-wife. In Excerpt 3, he relates his experience in a narration (Edwards and Potter, 1992). He delivered his narration in a streams-of-consciousness manner, which lends credibility to his experience because it would appear as a genuine recollection of events as opposed to elaborate reconstruction that is designed to promote a particular agenda.

#### Excerpt 3

**Table 3:** 

1.	Muzz:	We became close- close- close- we can- *err* what ya- yes until we
2.		got into romantic relationship. *Hmm* we were in love and all that.
3.		that because we were in long-distant relationship, from the
4.		day we got into relationship until marriage, we were in long distant
5.		relationship. So the problem had not really surfaced because we
6.		were far from each other. But it surfaced when we got married.
7.		*Haaa*. When we got married- when we got married- when we lived
8.		far from each other, all kinds of trouble surfaced. When there were
9.		a lot of problem, we became fed up with the relationship, this,
10.		everything became wearisome, everything would lead to argument.
11.		*Haaa* until one day, that time, we already had a child *lah*, the child
12.		was around 5–6 months like that. Wife- my ex-wife was like, was
13.		like suspicious *la*. How come she- how come I could- could stand
14.		not being with her for so long right. We would at least- because she
15.		worked in Sarawak- I worked in Sabah right at the time- *haa* – we
16.		met once a year. And even that, we met at the hometown, so she
17.		was suspicious right, that I could stand all that. Finally- but during
18.		that time- that time we were fighting *la*. She accused me of having
19.		another woman. We were fighting and fighting, and finally I came
20.		clean to her, that I am actually *err* gay and all, right. So, initially she
21.		was shocked at first then she said she was okay, she could accept
22.		it. But she wanted me to promise her *errr*- asked me to change.
23.		Can no longer do all those things. Right? But, I am only a normal
24.		human, right. I could not promise her that. I told her, I cannot. When-
25.		actually even during my marriage, it’s less. There were. But not-
26.		what people say- not as active as before. Sometimes, in 3, 4
27.		months’ time, only once, like that. Even that happened
28.		accidentally. I could not hold any longer, *haaa* only then would I did
29.		it. But since she asked me to promise to leave those things, I
30.		could not make the promise because I knew how I was and finally-
31.		finally- okay *lah* since no promise were made, she was willing to
32.		separate. She did not want to bear the risk. If anything happened,
33.		like sickness and all, right. So, we separated.

For this part, we examine how Muzz framed his infidelity. One striking part of his narration is how he only explicitly revealed that he had been cheating on his wife at a later part of his narration “actually even during my marriage, it’s less” [line 25]. Before this, he only alluded that he had been cheating on her “So the problem had not really surfaced” [line 5], and “how come I could- could stand not being with her for so long right” [line 13 and 14]. Here, Muzz applied Systematic Vagueness (Edwards and Potter, 1992) whereby his accounts would be less susceptible to be questioned due to its ambiguity. By not talking about his infidelity in a certain, blatant term, it also has the effect of minimizing the gravity of his action. It also has the effect of normalizing it. The way Muzz relates to his cheating behavior, it is as if the behavior is expected of gay men who get into straight marriages “But, I am only a normal human, right” [lines 23 and 24]. Ultimately, what this does is it attempts to absolve himself of accountability. Finally, the framing of his actions as merely an accidental happening “Even that, happened accidentally” [lines 27 and 28] and an event that happened not that often “Sometimes, in 3, 4 months, one time, like that” [lines 26 and 27] further compounds this notion.

### Attachment to Islamic Faith

For Malays, the Islamic faith plays significant roles to their individual, social, and national conscience. In the Malaysian Constitution, the Islamic faith is even deemed as indispensable of what constitutes an individual to be a Malay. Religion wise, Ahmad et al. (2017) highlighted that for a Muslim to participate in unlawful sexual activities, they are considered as weak spiritually and mentally as they are not fully conscious of the existence of Allah and do not consider the long-term effect of their actions in this world and the next. Thus, this part addresses how both participants’ Islamic faith informs their discourse on their lived experience as GMiSM.

In Excerpt 4 below, Fahrin responds to the interviewer’s line of questioning around the topic of guilt.

#### Excerpt 4

**Table 4:** 

1.	Interviewer:	But when you do those things, sexual relations with man, how do
2.		you feel after that? Do you- [Laughs] don’t know *la*, because sometimes it’s like I don’t really like doing se- se- sex because I am more into looking for someone
3.	Fahrin:	whom I can share- share problems with. Whatever problems I have
4.		right. Like that. but sometimes whe- when we meet a person aaa
5.		they sometimes have feelings towards me right aaa so sometimes-
6.		I do not know- that for me, I’m okay if I’m not being seduced but if I
7.		am being seduced, it’d be difficult a bit [laughs]. If people do not
8.		seduce me, then I can avoid the thing from happening, the sex I
9.		mean. In terms of satisfaction, everybody who has sex will normally
10.		feel satisfied right. It’s just that, actually.
11.		Do not you feel guilty?
12.		The guilt- maybe because I’m used to it, so I do not feel that much
13.	Interviewer:	guilt. Maybe if it is for the first- first time, maybe I’d feel guilty. I
14.	Fahrin:	mean for those who have just started to do it or for first timers or
15.		something like that. Having sex with man for example right. As for
16.		me- maybe for me - I mean this thing is normal.
17.		What about the fact that you are married?
18.		What’s that?
19.	Interviewer:	You’re married. Because you are married so do not you feel guilty
20.	Fahrin:	towards your wife? Or is the feeling-
21.	Interviewer:	I do not know, I do not really feel guilty [laughs].
22.		Is it because you do not have any feelings towards her? Cause you
23.	Fahrin:	said-
24.	Interviewer:	I- I do care about her but I am not in love with her. Because my
25.		attraction towards men is stronger than towards women, right
26.	Fahrin:	[laughs]. I like men more. And then about the guilt, you know-
27.		but I do feel- I mean sometimes I do think- like I do think as well,
28.		sometimes- I mean it’s not that I feel guilty towards my wife but I do
29.		feel guilty towards god, maybe. It’s like for other things, I have- I
30.		mean I have fulfilled other obligations like marriage, prayers and all
31.		those things but this thing- it’s difficult- to aaa- to what- to leave
32.		right. Sometimes the guilt is there but that’s about it.

Potter and Hepburn (2005) cautioned researchers to not treat interviewer’s part of the discourse as infallible so that even their part in the discourse ought to be scrutinized. Hence, here, we highlight the interviewer’s role in this discourse where it is apparent that the interviewer indeed has got a presumption that Fahrin ought to feel guilty for cheating on his wife. Admittedly, the line of questioning is problematic in that it subtly prompts Fahrin to answer in a particular way, i.e., he ought to feel guilty for cheating on his wife. Interestingly, however, the way Fahrin responds to the question did not go the way the question leads him on. Instead, we have got an incredibly transparent respond from Fahrin as he doubled down on his earlier position regarding his inculpability in his infidelity. In lines 9, 10, and 11, “If people do not seduce me, then I can avoid the thing from happening, the sex I mean” he framed himself as merely a less-than-willing participant in his sexual activity with other men. He also employed Consensus and Corroboration (Edwards and Potter, 1992) whereby his actions are framed as something normal, expected, and even agreeable to some extent. Case in point, he said that “this thing is normal” [Line 18] when citing the reason why he does not feel guilty for his actions. He also employed such expressions as “It’s just that, actually” [line 12] when talking about the pleasure of sexual intercourse; and “but that’s about it” [line 34] when talking about the lack of guilt he experienced. These expressions of excuse work well in further minimizing his act of infidelity.

Concerning Islamic expression, on the other hand, we can see it features prominently in lines 30 and 31 “I mean it’s not that I feel guilty towards my wife but I do feel guilty towards god, maybe.” Here, Fahrin acknowledged that his act of indulging in homosexual pleasure is indeed a sin. He also cited reason of why he feels so, “I have fulfilled other obligations like marriage, prayers, and all those things but this thing- it’s difficult-” [lines 32 and 33]. From this, it can be inferred that Fahrin is a Muslim who is diligent in carrying out the religious commands like marriage, five daily prayers, and fasting; he regards his inability to cease from leaving his homosexual tendency as the only thing that is hindering his spiritual fulfillment. Indeed, it is a common struggle for homosexual individuals with attachment to their religions that disapprove homosexuality to reconcile their faiths with their way of life. However, a supposedly “practicing” Muslim man would resort to such promiscuity without any regards to his wife certainly highlights the incongruence between the belief and the behavior of GMiSM of faith. The last thing that would be addressed for Excerpt 4 is the laughter that punctuates this part of this conversation [lines 3, 9, and 23]. Laughter could be studied in the context of its social and discursive function. Fahrin’s laughter here is not because he finds the situation particularly humorous as was noted through our memoing that he appeared uncomfortable when he laughed. Thus, how his laughter punctuated his discourse when he was posed with a rather tricky question by the interviewer ought to be seen as a more complex discursive coping strategy, i.e., he was trying to cope with how he would appear disclosing his behavior as well as his own possibly subconscious guilt. Partington (2009) states that laughter could be used to signal embarrassment. We can see this in the context of Fahrin’s discourse. Although Fahrin constructed his discourse in such a way that he appears unremorseful of his action, his laughter which occurs while he was talking about this challenging topic, indicated that he was uncomfortable and understood how he would look responding to the questionings by the interviewer. Hence, Fahrin’s laughter serves a substantial purpose in that it signals that Fahrin did feel guilt, albeit it was expressed subconsciously.

For Muzz, he related his experience of when he fell sick due to complications from HIV and how this experience initiated his developing connection back to his Islamic faith. Before the conversation in Excerpt 5, Muzz was relating how his “wild” lifestyle after his marriage ended led him to getting HIV.

#### Excerpt 5

**Table 5:** 

1.	Interviewer:	You were hospitalized due to leptospirosis? Leptospirosis infection?
2.	Fahrin:	Initially- initially, they diagnosed it as leptospirosis. But at the time it
3.		did not seem to get better. It had been a week but it did not get better.
4.	Interviewer:	It did not get better… because of HIV?
5.	Fahrin:	*Haa* cause of that. Because of HIV. So when… when they took-
6.		they took- er, what’s that called- took fluid from my spine, then they
7.		checked. And then they- they- er the doctor expected- they did HIV
8.		test. *Haa* only then we knew. After the check up, everything at that
9.		time was already low. *Haa* okay. It’s just that when- when it came to
10.		that-
11.	Interviewer:	That was in 2000-?
12.	Fahrin:	Diagnosed in 2017.
13.	Interviewer:	Ooo… two years ago. So this was recent.
14.	Fahrin:	*Haa* only recently. So when it came to that, it really- at the time- I was
15.		really- I was really- when I fell sick right. That’s what lead to my
16.		[spiritual] awakening *lah*, that’s what people call it right. Actually,
17.		when I’d got sick, that really made me realize *lah*. Err there was this
18.		one- one- it actually already felt like dying. At the time, I felt like I was
19.		on the verge of death. With no preparation- and then people back
20.		home had started to recite the *Yaasin* and all. My condition, it was
21.		like I was left with an inch of my life *la* as people say. At the hospital.
22.		*Haa* but at the time I was conscious, I was conscious that people were
23.		visiting, people were reciting the *Yaasin* for me and all that. But I
24.		could not open my eyes, could not wake up, could not move at all. But I
25.		could hear, could hear everything right. At the time I was really like
26.		half-conscious *la*. It felt like O Allah if You really want to take my life
27.		in my [sinful] state like this, then please do so. But if You really want
28.		to give me the chance to change, then grant me the strength to
29.		change, right. At the time, I could really feel, right, in that state of
30.		helplessness, not one single thing could help us at the time, no matter
31.		if it’s our money, or our family, or our children, nobody, would be with
32.		us- nobody could help us at the time. I was heedless. There was this
33.		one time I felt like my life was about to be taken. But I wanted to
34.		profess [the *syahadah*] and I did not know how, Everything I wanted to
35.		do, I did not know how to do. I was delirious on my bed. Like,
36.		thought I was dying that I asked my mom how to profess the
37.		*syahadah* but my tongue could not utter it. O Allah I was so bad, how
38.		could I let myself be like this?

In this excerpt, Muzz provides a rather Vivid Description (Edwards and Potter, 1992) of a Narrative (Edwards and Potter, 1992) retelling of his experience. These discursive techniques are effective measures in establishing one’s credibility. When Muzz employed Vivid Descriptions of what he experienced when he was in comatose, “I was conscious that people were visiting, people were reciting the *Yaasin* for me and all that. But I could not open my eyes, could not wake up, could not move at all.” [lines 22–24], he makes his experience feel real to his audience, and this would certainly make his account seems truthful. On an emotional level, the sense of helplessness he conveyed through the description of his auditory experience, i.e., only being able to merely hear what people around him did without being able to do anything, would make the audience see how brutal the disease ailed his body. His reconstructing of his experience in such a structured narrative also helps in advancing his agenda. He chose to highlight a moment of realization “At the time, I could really feel, right, in that state of helplessness, not one single thing could help us at the time, no matter if it’s our money, or our family, or our children, nobody, would be with us- nobody could help us at the time.” [lines 29–32]. He also acknowledged that he was “heedless” [line 32] of God’s command. This part in his narration represents a pivotal moment that moved Muzz toward repentance. But what is his agenda exactly? Here, we argue that the Action (Edwards and Potter, 1992) that Muzz was trying to achieve through his discourse is to make his story serves as a reminder for Muslims not to embrace homosexuality. The inclusion of monologues “O Allah if You really want to take my life in my [sinful] state like this, then please do so. But if You really want to give me the chance to change, then grant me the strength to change” [line 26–29] and “O Allah I was so bad, how could I let myself be like this?” [lines 37 and 38] certainly make his story emotionally charged, and this further strengthens the earlier notion that Muzz was employing his experience as a way to do proselytizing against homosexuality.

## Discussion

In this section, the findings would be further explicated in a broader societal context. It is undeniable, especially in the case of Fahrin, that the way they present their discourse paint them in a negative light. To simply vilify them however is not enough to address the issue, rather, it would be more productive if we shift the focus of the examination toward the society in which the phenomenon of GMiSM cheating on their wives seems to occur without impunity. The two cases we examine and explicate through the subjects’ spoken discourse provide valuable insight into the lives of GMiSM. What is revealed through their discourse is definitely problematic; while the lack of remorse that is captured through DP and DAM analysis provides elucidating insight into the intricacies of such relationships.

For Muslims, even affirming that act of homosexuality as anything other than sin is a sin in itself. Doing so would mean that the Muslims deny the “words of Allah” and thus, their faith would be compromised. Islam also put great emphasis on not airing one’s (of yourself or others’) sinful behavior to everyone. These, according to Ali (2006), create a ripe environment for the matter to be swept under the rags rather than be openly addressed. The media role in Malaysia in exacerbating the issue is also apparent. Homosexuality is often portrayed in a simplistic term whereby homosexuals are deemed as repulsive and disgraceful (Shah, 2018). Henceforth, it is only natural that Muslim society, in general, lacks a nuanced understanding of this matter. The stigma attached to the perceived unusual nature of homosexuality makes it unthinkable for a lot of Muslims to fathom if their family members or friends are gay. This willful denial aggregates the matter further as gay men are pressured to conform to heteronormative Islamic societal norm and get married.

Consequently, it is also absolutely crucial for Muslim society to have a more comprehensive, national conversation on the issue. To do this, let us look into the past. In canonical work of El-Rouayheb (2009) on homosexuality in the Islamic Arab society during the late Ottoman Period, he extensively chronicled how homosexuality (back then it was often characterized as the desire of adult men toward young, “beardless” teen) was normalized in the society. Affectionate love toward boyish teens was not only considered as inevitable, but it was also celebrated in poetry. So widespread was such behavior that there was even specialized poetic genre dedicated solely for “love towards male youths.” The poems range from being outright lewd “He prized open the boy’s ass with the edge of his “sword,” then pierced him to the hilt with the head of his “lance” (Abu Nuwas, al-Nusus al Muharrama as cited in Kennedy (2012); to tame ones that merely addressed the poet’s chaste infatuation),

He’s a radiant moon if he appears; a succulent branch if he sways.

He looks with a gazelle’s eyes, but fills my heart with fear and trembling.

By God! By God! Have mercy upon me, O wispy shape!

Yearning has melted me, and undone the knot of patience.

What is the fault of my heart that it should be ever in flames?

(Nabulusi, in El-Rouayheb, 2009, p. 104).

It needs to be emphasized however that homosexuality was never condoned by the Islamic religious institutions and scholars even during that time when it was normalized. Many religious scholars even prohibit adult men from being alone with young men or even gazing too long at them the same way it is prohibited to do so with women. Then, the issue of homosexuality was publicly debated, and rulings or opinions regarding the issue were extensively recorded. As a result, there exists a phenomenon where men of prominent stature made their homosexuality be known to the public. One such figure is Abdallah al-Shabrawi who held the position as Rector of the most prestigious college in the Muslim world, the Al-Azhar’s college, for over 30 years. He was well known for producing a collection of love poems toward his “male beloveds.” However, although he commemorated his infatuations toward boys in poetry, he also repeatedly stressed that his predisposition toward males was not something he wished to indulge; his love for them was no more than a chaste desire. Moreover, indeed, many religious scholars of the time even held the opinion that “a person who died from unconsummated love for a boy could earn the status of a martyr (*sha- h ¯ıd*), which would guarantee him a place in heaven” (El-Rouayheb, 2009, p. 139).

What work of El-Rouayheb (2009) illustrates is how homosexuality within a society could exist in the realm between openness and prohibition, between piety and carnal desire, and between the sacred and the sacrilegious. The conception of homosexuality portrayed in his work was nothing like what it is today in the Western world nor the Islamic world. Hence, what this presents are an alternative of how a society could operate within the boundary of desire and prohibition. Although, of course, we are not advocating for the Islamic society to emulate the how Islamic empire in the past treated homosexuality. What we are suggesting, however, is that the matter should not be a taboo topic. Comprehensive sex education covering the LGBT spectrums backed by reputable science and informed by works from gender and sexuality studies would be fundamental in tackling the issue.

Other than that, this research also highlights the misogyny perpetrated by GMiSM toward their wives. This is an interesting phenomenon. As gay men in this country are certainly marginalized, we could see how their position as men would still allow them to be in the position of power to oppress women. In Fahrin’s case, his wife’s apparent obliviousness of Fahrin’s infidelity certainly put her at a position of disadvantage. Other than imminent mental distress that would be born from such marriage, the physical harm that could come from gay husbands’ risky sexual behavior also poses a real threat toward women in such marriages. For Muzz’s spouse, on the other hand, we could infer from her willingness to forgive and accept Muzz even after she found out about Muzz’s infidelity that she was also conditioned to accept such a disadvantageous position in her marriage. The two cases presented in this research illustrate the stark reality of the oppression faced by women who are married to gay men.

Finally, the participants’ sense of attachment to their Islamic faith is also a significant aspect of this study. Given that Islamic identity is such an integral part of Malays identity capturing the intertwining of the participants’ “illegitimate” sexuality and ensuing infidelity with the convictions of their faith through their spoken discourse would be crucial in providing novel insight into the phenomenon of GMiSM in the Malay-Muslim context. Through DP and DAM, we manage to explicate how Muzz performed his religiosity in the “Narrative” (Edwards and Potter, 1992) of the event that led to his repentance and how this coupled with “Vivid Description” (Edwards and Potter, 1992) worked to further push heteronormative Malay-Muslim hegemony in Malaysia. Fahrin also succumbs to the same heteronormative ideal. He acknowledges that engaging in gay sex is definitely forbidden in Islam, as well as practices Islamic obligations diligently. This is in stark contrast to the way he presented his discourse regarding his infidelity, where it was presented as if he was inculpable for his cheating; instead, he put the blame on his wife, who he deems as lacking in living up to his expectation of how a wife should be. Therefore, this research manages to highlight the disconnection between Fahrin’s aspired Islamic ideal and his actual lived reality.

The notion of Discourse as Action is also an important aspect to look into. For Muzz, he utilized the opportunity to tell his story in the interview process as a way of reaffirming his stance against homosexuality despite him being a homosexual himself. The delivery of the horror he faced when he almost died of HIV-related complications served as a rather compelling precautionary account from a repentant homosexual of why his fellow gay Muslims should not embrace homosexuality. For Fahrin, on the other hand, his account of his infidelity was foregrounded. The blatant blaming of his wife provides a rather illuminating cognizance into GMiSM’s psyche. From Fahrin’s discourse, we can see how Fahrin distanced himself from the gravity of his action by maneuvering his discourse to deflect accountability and blame toward his wife. The nonchalant attitude he assumed concerning his unfaithfulness is another crucial aspect that ought to be addressed, i.e., what drives Fahrin to be so transparent with his feeling? We opined that this reflects the prevalence of GMiSM cheating on their wives so that Fahrin felt that what he expressed was neither shocking nor obscene.

## Conclusion

As the literature suggests, the prevalence of infidelity among GMiSM causes significant problems toward the spouses as well as a great conflict to such relationships. Hence, this study provides further nuanced insights into the psyche of GMiSM, particularly on their infidelity. Through DP and DAM, we elucidated on account of blame and accountability when they talk about this topic. The role of the subjects’ Islamic faith is also explicated vis-à-vis their sexuality and infidelity. The struggle of Fahrin and Muzz in grappling with their sexuality in light of their faith is common among homosexual Muslims in Malaysia (Shamsudin and Ghazali, 2011; Jerome, 2013; Zulkffli and Rashid, 2019). For Fahrin, his inability to do away with his infidelity despite describing himself as a practicing Muslim and acknowledging that it is wrong is indicative of how religious and social institutions in Malaysia fail to address the LGBT dilemma adequately. Muzz, on the other hand, uses the interview process as a way to relay his narrative on how he came about to his repentance. It is crucial to note that the two subjects subvert and reject the conception of sexuality as it is commonly advocated in the Western world. All in all, as this research only addresses GMiSM, through two cases, further research with more subjects and multifaceted approaches are needed to provide a complete picture of the phenomenon of GMiSM.

## Data Availability Statement

The original contributions presented in the study are included in the article/supplementary material, further inquiries can be directed to the corresponding author.

## Ethics Statement

Ethical review and approval was not required for the study on human participants in accordance with the local legislation and institutional requirements. The patients/participants provided their written informed consent to participate in this study.

## Author Contributions

MZ and RA conceived the original idea. MZ carried out the research. RA helped to supervise the research. MZ, RA, MK, and HI, wrote the manuscript. All authors contributed to the article and approved the submitted version.

## Conflict of Interest

The authors declare that the research was conducted in the absence of any commercial or financial relationships that could be construed as a potential conflict of interest.

## Publisher’s Note

All claims expressed in this article are solely those of the authors and do not necessarily represent those of their affiliated organizations, or those of the publisher, the editors and the reviewers. Any product that may be evaluated in this article, or claim that may be made by its manufacturer, is not guaranteed or endorsed by the publisher.
